# Spider angiomas as an unusual cutaneous clue to very early systemic sclerosis

**DOI:** 10.1016/j.jdcr.2026.08.031

**Published:** 2026-09-01

**Authors:** Ines Lederbogen, Barbara Meier-Schiesser

**Affiliations:** a Department of Dermatology, University Hospital Zurich, Zurich, Switzerland; b Medical Faculty, University of Zurich, Zurich, Switzerland

**Keywords:** Raynaud phenomenon, spider angiomas, systemic sclerosis, telangiectasia

## Introduction

Unilateral or segmental spider angiomas are uncommon and are most frequently associated with unilateral nevoid telangiectasia, hormonal influences, pregnancy, or hepatic disease.^1^^,^^2^ These associations are typically well-recognized and may lead to attribution of the lesions to hormonal or hepatic causes, potentially obscuring alternative underlying diagnoses. In contrast, telangiectasias in systemic sclerosis (SSc) usually present as bilateral and widespread lesions that increase with disease duration and reflect progressive microvascular dysfunction. The occurrence of unilateral or segmentally distributed spider angiomas in this context is highly unusual and may delay recognition of an underlying connective tissue disease.

We report a patient with unilateral, segmentally distributed spider angiomas as an unusual vascular presentation associated with very early SSc.

## Case report

A 27-year-old pregnant woman presented with segmentally distributed spider angiomas, initially localized to the right scapular region (Fig 1, *A*). The lesions were blanchable, with a central arteriole and radiating telangiectatic vessels, and showed no central atrophy, ulceration, or scarring. During pregnancy, the lesions increased in number and extent, involving the trunk, extremities, hands, and lips (Fig 1, *C*). The close temporal association with pregnancy initially suggested a hormonally mediated vascular process.

**Fig 1:**
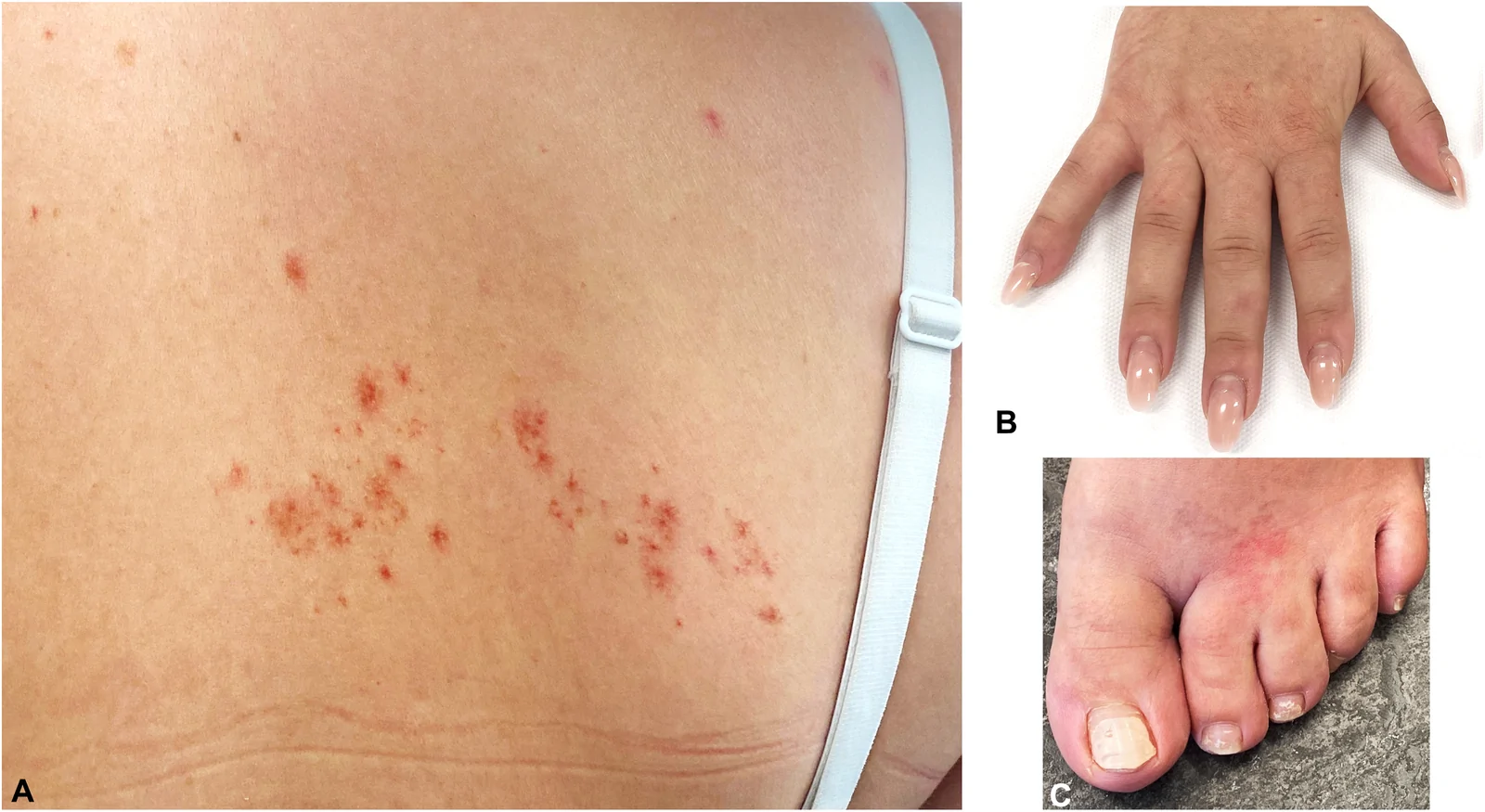
Clinical appearance of **(A)** atypical segmentally distributed spider angiomas on the trunk, **(B)** edematous fingers (puffy fingers), and **(C)** a later development of a spider angioma on the left dorsal foot.

Raynaud phenomenon had been present since age 19, with slowly progressive uniphasic color changes of the fingers and toes and no history of digital ulcers. As the symptoms were mild and non-disabling, they had not previously prompted medical evaluation. Physical examination revealed edematous hands without skin thickening, sclerodactyly, pitting scars, or calcinosis (Fig 1, *B*). She denied dyspnea, chest pain, or dysphagia but reported intermittent gastroesophageal reflux. Hormonal contraception had been discontinued several years prior to symptom onset. Family history was negative for autoimmune or rheumatic disease.

Given the combination of Raynaud phenomenon, gastroesophageal reflux, and atypical vascular lesions, serologic testing was performed. Antinuclear antibodies were strongly positive at a titer of 1:10,240 with a centromere fluorescence pattern. Anti-centromere A and B antibodies were markedly elevated. This antibody pattern is strongly associated with limited cutaneous SSc and vascular manifestations. Liver function tests and thyroid-stimulating hormone levels were within normal limits.

Although the patient did not meet the full 2013 ACR/EULAR classification criteria for SSc due to the absence of skin fibrosis, the combination of long-standing Raynaud phenomenon, disease-specific autoantibodies, and vascular skin findings was considered indicative of very early SSc. The pregnancy was otherwise uncomplicated and resulted in the delivery of a healthy infant. The infant showed no clinical features suggestive of neonatal lupus. In the absence of clinical suspicion, serological evaluation of the infant was not performed.

Postpartum, the patient underwent cardiopulmonary screening, including transthoracic echocardiography, which showed a small pericardial effusion but no evidence of pulmonary arterial hypertension. Pulmonary function testing demonstrated normal forced vital capacity.

Over a follow-up period of several years, the disease course remained stable, with a mild clinical phenotype and no evidence of progressive skin fibrosis or internal organ involvement. Management focused on symptomatic treatment and longitudinal monitoring.

Raynaud phenomenon was managed with cold avoidance and later amlodipine. Gastroesophageal reflux was treated with proton pump inhibitors, and dietary modification was recommended for suspected small intestinal bacterial overgrowth. Given the cosmetic impact of the vascular lesions, laser therapy was discussed as a treatment option; however, the patient declined interventional therapy at the time of reporting.

## Discussion

Cutaneous vascular abnormalities may provide an important clue to underlying SSc.

SSc is characterized by immune-mediated vasculopathy and progressive fibrosis. Telangiectasias are a characteristic cutaneous feature and typically present bilaterally, increasing with disease duration and reflecting progressive microvascular damage. They are part of the clinical features of limited cutaneous SSc, historically summarized by the CREST acronym, which includes calcinosis cutis, Raynaud phenomenon, esophageal dysmotility, sclerodactyly, and telangiectasias.^3^, ^4^, ^5^

Reports of unilateral, segmentally distributed spider angiomas in association with SSc are exceedingly rare, particularly as an unusual vascular presentation in patients without established skin fibrosis.

Unilateral, segmentally distributed spider angiomas may represent an uncommon vascular finding associated with very early SSc.

In patients with atypical vascular skin lesions, particularly when accompanied by Raynaud phenomenon and gastroesophageal reflux, serologic evaluation may be considered to enable early diagnosis and appropriate surveillance. This case underlines the importance of maintaining a high level of suspicion for autoimmune connective tissue disease in patients presenting with atypical or unilateral vascular skin lesions.

### Declaration of generative AI and AI-assisted technologies in the writing process

Not applicable.

## Conflicts of interest

None disclosed.
